# Cloning and expression of *Staphylococcus simulan*s lysostaphin enzyme gene in *Bacillus subtilis* WB600

**DOI:** 10.3934/microbiol.2021017

**Published:** 2021-07-23

**Authors:** Babak Elyasi Far, Mehran Ragheb, Reza Rahbar, Ladan Mafakher, Neda Yousefi Nojookambari, Spyridon Achinas, Sajjad Yazdansetad

**Affiliations:** 1 Department of Pharmaceutical Biotechnology, Faculty of Pharmacy, Tabriz University of Medical Sciences, Tabriz, Iran; 2 Student Research Committee, Golestan University of Medical Sciences, Gorgan, Iran; 3 Medical Plant Research Center, Ahvaz Jundishapur University of Medical Science, Ahvaz, Iran; 4 Department of Microbiology, School of Medicine, Shahid Beheshti University of Medical Sciences, Tehran, Iran; 5 Faculty of Science and Engineering, University of Groningen, Groningen, The Netherlands; 6 Laboratory Sciences Research Center, Golestan University of Medical Sciences, Gorgan, Iran

**Keywords:** *Bacillus subtilis*, Lysostaphin, *Staphylococcus aureus*, plasmids, enzyme stability

## Abstract

Lysostaphin is a glycylglycine endopeptidase, secreted by *Staphylococcus simulans*, capable of specifically hydrolyzing pentaglycine crosslinks present in the peptidoglycan of the *Staphylococcus aureus* cell wall. In this paper, we describe the cloning and expression of the lysostaphin enzyme gene in *Bacillus subtilis* WB600 host using pWB980 expression system. Plasmid pACK1 of *S. simulans* was extracted using the alkaline lysis method. Lysostaphin gene was isolated by PCR and cloned into pTZ57R/T-Vector, then transformed into *Escherichia coli* DH5α. The amplified gene fragment and uncloned pWB980 vector were digested using *Pst*I and *Xba*І enzymes and purified. The restricted gene fragment was ligated into the pWB980 expression vector by the standard protocols, then the recombinant plasmid was transformed into *B. subtilis* WB600 using electroporation method. The recombinant protein was evaluated by the SDS-PAGE method and confirmed by western immunoblot. Analysis of the target protein showed a band corresponding to 27-kDa r-lysostaphin. Protein content was estimated 91 mg/L by Bradford assay. The recombinant lysostaphin represented 90% of its maximum activity at 40 °C and displayed good thermostability by keeping about 80% of its maximum activity at 45 °C. Heat residual activity assay of recombinant lysostaphin demonstrated that the enzyme stability was up to 40 °C and showed good stability at 40 °C for 16 h incubation.

## 1. Introduction

Lysostaphin is a zinc-metalloprotease glycylglycine endopeptidase enzyme originally secreted by *Staphylococcus simulans biovar staphylolyticus*. Lysostaphin specifically disrupts pentaglycine crosslinks of peptidoglycan, probably between the third and fourth glycine residues, in the *Staphylococcus aureus* cell wall [1]–[3]. Interestingly, lysostaphin able to lyse cells in all metabolic conditions, including growing, resting, and heat-killed [4]. Lysostaphin is encoded by the lysostaphin endopeptidase gene (lss) which is present on plasmid pACK1, a large β-lactamase plasmid with a size of 55171 bp, belonging to the *S. simulans* [5]. Lysostaphin is initially produced as a preproenzyme of 493 amino acids with three domains: an N-terminal domain as the secretion signal peptide of 36 amino acid residues, a proenzyme of 211 amino acid residues harboring 15 tandem repeats (TRs) of 13 amino acids, and a mature enzyme of 246 amino acid residues. The signal peptide is intracellularly cleavaged and propeptide detached extracellularly by a cysteine protease to yield the mature and activated lysostaphin [1],[6]. The mature lysostaphin is 4.5-fold more active rather than the prolysostaphin. The mature lysostaphin consists of two domains: an N-terminal peptidase domain involved in the catalytic activity of the enzyme, and a C-terminal domain binding to the peptidoglycan [1]. Previous studies reported that the C-terminal domain with 92 amino acids not involved in the enzymatic activity; however, it plays an important role in directing lysostaphin to the *S. aureus* cell wall [2]. Lysostaphin is produced in the stationary-phase cultures of *S. simulans* and it seems to be synchronized with the production of other extracellular enzymes (i.e., proteases and hexosaminidases) [7],[8]. The molecular weight, p*I* (isoelectric point), and pH optimum of lysostaphin are about 27 kDa, 9.5, and 7.5, respectively [5]. Lysostaphin exhibits a broad spectrum of anti-staphylococcal properties making it a promising candidate for numerous biotechnological applications in the fields of medical, veterinary, food industries, and researches[9],[10]. Significantly, lysostaphin can kill the multidrug-resistant strains of staphylococci especially methicillin-resistant *S. aureus* (MRSA) and vancomycin-resistant *S. aureus* (VRSA) [11]. On the other hand, lysostaphin is effective in lysing some other staphylococci species such as *S. epidermidis*, *S. haemolyticus, S. lugdunensis*, and *S. saprophyticus* [12]. Lysostaphin also degrades glycine-rich proteins like insoluble elastin [13]. There are some interesting features of lysostaphin making it a unique therapeutic agent, including its activity against non-dividing as well as dividing cells, its digestion by intestinal proteinases, not influence the gut microbiota, non-toxicity, its relative stability in conjugating with polyethylene glycol (PEG), and maintenance of its activity in human serum [1],[3]. Furthermore, it has been demonstrated that the lysostaphin preserves its bacteriolytic activity *in vivo*, without any unwanted immune reactions, despite the presence of a high-neutralizing antibody titer [14]. To overproduce the recombinant lysostaphin and to purify from a safe and nonpathogenic source, we selected *Bacillus subtilis* as an expression host with high capability in protein secretion. In the present study, we examine the cloning and expression of the *S. simulans* lysostaphin enzyme gene in *B. subtilis* WB600 using expression vector pWB980. The recombinant product can be safely used as a novel antimicrobial agent in the treatment of staphylococcal infections.

## 2. Materials and methods

### 2.1. Bacterial strains, plasmids, enzymes, and reagents

**Figure 1.**
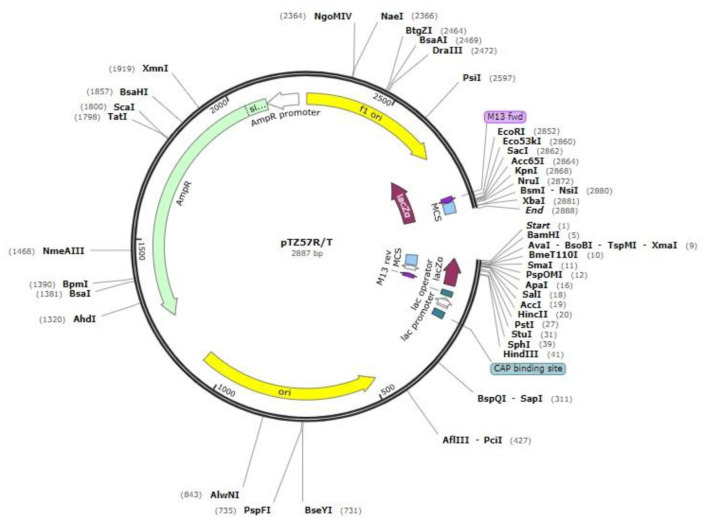
Map of pTZ57R/T vector (SnapGene^®^).

**Figure 2.**
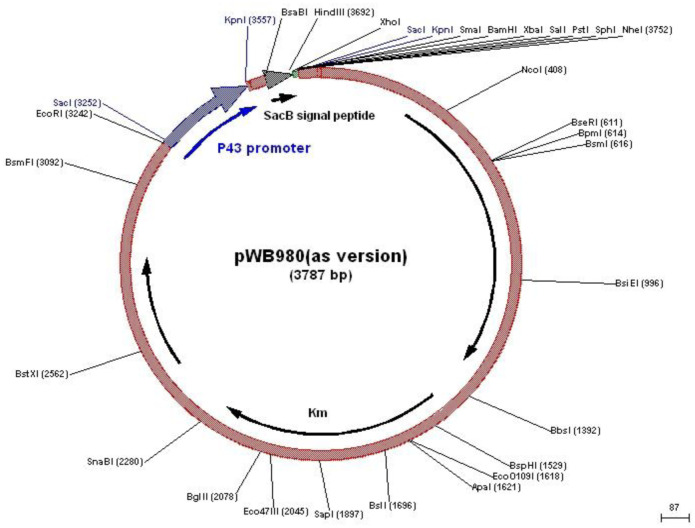
Map of pWB980 vector (Life Science Market)

*Staphylococcus simulans biovar staphylolyticus* (ATCC 27848), as the source of the lysostaphin gene, was obtained from Persian Type Culture Collection (PTCC, Tehran, Iran). *E. coli* DH5α was purchased from Novagen Company (Novagen, Madison, WI, USA). *Bacillus subtilis* strain WB600, the bioengineered strain deficient in six extracellular proteases[15], was kindly provided by Professor Sui-Lam Wong (University of Calgary, Canada). The pTZ57R/T vector (Figure 1) provided by Fermentas Co., Ltd (Fermentas, Vilnius, Lithuania). The expression vector pWB980 (Figure 2) was prepared from Nova Lifetech Inc., Hong Kong. The restriction enzymes *Pst*I, *Xba*І and *Eco*RІ were purchased from Vivantis (Vivantis, Malaysia). T4 DNA Ligase, *pfuTurbo* DNA polymerase, and *Taq* DNA polymerase enzymes were bought from GeneON (GeneON, Nurnberg, Germany), Fermentas (Fermentas, Vilnius, Lithuania), and Cinnagen (Cinnagen, Tehran, Iran), respectively. Oligonucleotide primers were synthesized by TAG Copenhagen, Denmark. DNA Ladder O'GeneRuler 1 kb Plus was purchased from Thermo Scientific™. Broad range unstained protein standard marker was provided by Fermentas Co., Ltd. All culture media were provided by HiMedia Laboratories Pvt. Ltd., India.

### 2.2. Plasmid extraction and PCR cloning of lysostaphin gene

The plasmid was extracted from *S. simulans* using an optimized alkaline lysis method. Briefly, the bacterial cell pellet was resuspended in 100 µL resuspension buffer (Tris-Acetat 40 mM, Sodium-EDTA 2 mM, pH 7.9), then the cells were lysed with 200 µL lysis solution (Tris 50 mM, SDS 3%, pH 12.6) and incubated in a water bath at 65 °C for 15 min, and then neutralized with 450 µL of cold potassium acetate 5 M, glacial acetic acid, pH 4.8. The mixture was centrifuged at 13,000 rpm for 10 min at 4 °C. The supernatant was mixed with an equal volume of isopropanol and placed at −20 °C for 20 min. The mixture was centrifuged at 13,000 rpm for 10 min at 25°C. The supernatant was removed and 200 µL of 70% ethanol was added to the pellet and centrifuged at 13,000 rpm for 5 min at 25 °C. The pellet was resuspended in 50 µL dH_2_O. The extracted plasmid was visualized on a 1% agarose gel stained with ethidium bromide and run at 90 V for 50 min.

Specific primers for PCR amplification of the lysostaphin gene were designed according to the known lysostaphin gene sequence from *S. simulans* deposited in GenBank under accession No. M15686.

The forward primer (5′-AGATCTAGAGCTGCAACACATGAACATTCAGCA-3′), *Xba*І restriction site underlined in the primer sequence, and the reverse primer (5′-TCACTGCAGCTTTATAGTTCCCCAAAGAACAC-3′), *Pst*I restriction site underlined in the primer sequence, were used for PCR amplification of the lysostaphin gene. The PCR reaction mixture was prepared in a total volume of 25 µL containing 10 nanogram (ng) of genomic DNA, 10 mM dNTP mix, 10 pM of each oligonucleotide primer, and 2.5 U *Taq* DNA polymerase in PCR buffer with 20 mM MgSO_4_. The PCR reaction was carried out in thermal cycler Peqlab Primus 25 (Peqlab Primus 25, UK) under the following steps: 5 min initial denaturation at 94 °C, followed by 35 cycles of denaturation at 94 °C for 1 min, annealing at 58 °C for 1 min, synthesis at 72 °C for 1 min, followed by 10 min elongation at 72 °C. PCR product was electrophoresed on 1% (w/v) ethidium bromide-stained agarose gel and purified using GeneJET PCR Purification Kit (Thermo Fisher Scientific) according to the manufacturer's instructions.

### 2.3. Cloning of lysostaphin gene

The pTZ57R/T vector and *E. coli* DH5α were used for the lysostaphin gene cloning, pWB980 expression vector and *B. subtilis* WB600 were utilized for the subcloning. The ligation reaction was done between the PCR product ~600 ng and pTZ57R/T vector ~200 ng. The ligated vector was transformed into *E. coli* DH5α by cold CaCl_2_ shock method. Then, 100 µL of the transformed *E. coli* DH5α was cultured aerobically in Luria–Bertani (LB) medium supplemented with 100 µg/mL ampicillin, 30 µg/mL X-gal, and 2 mM IPTG at 37 °C for overnight. The white colonies on the agar medium were designated and subcultured, followed by the recombinant plasmids were extracted and analyzed. The lysostaphin gene fragment was digested by *Pst*I and *Xba*І enzymes from T-vector. The expression vector pWB980 was also digested by the same enzymes (*Pst*I and *Xba*І) and purified by GeneJET Purification Kit, then ligated to the pWB980 expression vector by the standard protocols.

### 2.4. Transformation into Bacillus subtilis and construction of expression vector

*B. subtilis* WB600 was used for extracellular production of recombinant lysostaphin. The ligation reaction was carried out with 1µg pWB980 plasmid and 3 µg gene fragment. Afterward, 20 µL of the ligation mixture was transformed into *B. subtilis* by electroporation method at 8 milliseconds and 950 V (Gene Pulser Xcell™ Electroporation System, Bio-Rad, USA). Finally, 200 µL of the transformed *B. subtilis* was aerobically cultured in LB medium supplemented with 10 µg/mL kanamycin (Sigma-Aldrich) at 37 °C for 16 h.

### 2.5. Expression of lysostaphin

*B. subtilis* cells with pWB980 plasmid encoding lysostaphin gene were grown in LB medium supplemented with 10 µg/mL kanamycin on a rotator shaker (180 rpm) at 37 °C to reach an optical density (OD) of 1.2–1.5 at a wavelength of 600 nm. The cells were centrifuged and the supernatant was fractionated by adding solid (NH_4_)_2_SO_4_ at 4 °C. The protein was pelleted and resuspended in phosphate buffer solution (0.1 M, pH 7.2).

### 2.6. SDS-PAGE and Western blotting

Sodium dodecyl sulfate polyacrylamide gel electrophoresis (SDS-PAGE) was achieved on 12% (w/v) resolving gel as described by Laemmli [16]. The recombinant protein band was monitored by using SDS-PAGE following Coomassie brilliant blue G-250 staining method. The protein concentration was measured by Bradford assay using bovine serum albumin (BSA) as the standard [17]. For the immunoblotting assay, a replicate gel was moved onto a nitrocellulose membrane (Macherey-Nagel™ Porablot NCP, Germany) for 1.5 h at 320 V, soaked in 5% BSA blocking solution for overnight at 4 °C, then washed 3 times by TBS-T (Tris-HCl 20 mM, NaCl 150 mM, pH 7.5-Tween 0.05%). The blots were incubated with a dilution of 1:100 antiserum, followed by a 1:2000 dilution of HRP- labeled Goat Anti-Rabbit IgG (SouthernBiotech, USA) in TBS-T for 1 h. The signals were developed with DAB (3,3′-Diaminobenzidine) (Sigma-Aldrich, USA) and H_2_O_2_ substrate.

### 2.7. Bacteriolytic activity of r-lysostaphin

The bacteriolytic activity of r-lysostaphin was assayed by spectrophotometric measurements of turbidity as previously described by Marova and Kovar [18]. Briefly, the reaction mixture, containing 6 ml suspension of *S. aureus* DSM 1104 diluted in PBS (Phosphate-buffered saline 0.1 M, pH 7.2) to reach OD_620 nm_ = 0.25, was preincubated at 37 °C for 10 min and then 20 mL of r-lysostaphin was added. The changes in turbidity of the reaction mixture were determined. One unit of lysostaphin activity was defined as a total preparation affecting 50% turbidity reduction of the bacterial cell suspension at absorbance of 620 nm (A620 nm) within 10 min at 37 °C in a 10 mm cuvette. The pH and temperature parameters on recombinant enzyme activity were assayed in the ranges of 5.0–10.0 and 20–60 °C, respectively. The residual activity of r-lysostaphin was also studied at different temperatures and time treatments.

## 3. Results

### 3.1. Cloning of lysostaphin enzyme gene in E. coli

**Figure 3.**
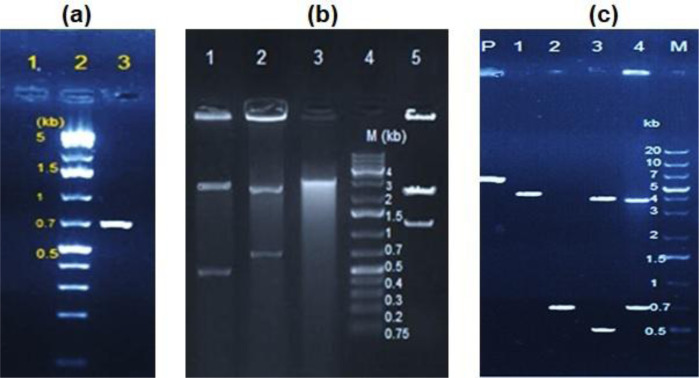
(a) Agarose gel electrophoresis analysis for PCR amplification of the lysostaphin gene. Lane 1: PCR negative control (NC); Lane 2: DNA Ladder O'GeneRuler 1 kb Plus; Lane 3: PCR product. (b) Agarose gel electrophoresis analysis for verifying lysostaphin gene orientation within the recombinant pTZ57R/T vector. Lane 1: Double digestion of plasmids with *Mro* NІ and *Xba*І indicating incorrect gene fragment orientation; Lane 2: Double digestion of plasmids with *Xba*І and *Pst*I; Lane 3: Single digestion of plasmids with *Xba*І; Lane 4: DNA Ladder O'GeneRuler 1 kb Plus; Lane 5: Double digestion of plasmids with *Mro* NІ and *Xba*І confirming the desired gene fragment orientation. (c) Agarose gel electrophoresis analysis for verifying lysostaphin gene orientation within the recombinant pWB980 vector. Lane P: Undigested plasmids; Lane 1: Single digestion of plasmids with *Eco*RІ; Lane 2: PCR product from recombinant plasmids; Lane 3: Double digestion of plasmids with *Eco*RІ and *Xba*І indicating desired orientation; Lane 4: Double digestion of plasmids with *Xba*І and *Pst*I; Lane M: DNA Ladder O'GeneRuler 1 kb Plus.

PCR amplification of the lysostaphin enzyme gene from *Staphylococcus simulans* showed an expected amplicon size of 744 bp (Figure 3a). Cloning of the lysostaphin gene fragment using pTZ57R/T vector in *E.coli* DH5α host strain resulted in formation of recombinant clones harboring the gene. The recombinant plasmids were digested to 2.9 kb and 0.7 kb segments by the *Xba*І and *Pst*I enzymes, and 2.4 kb and 1.2 kb segments by the *Mro* NІ and *Xba*І enzymes verifying the gene cloning and orientation properly (Figure 3b).

### 3.2. Construction of B. subtilis expression vector pWB980

Recombinant plasmid clones carrying the lysostaphin gene were double digested by *Xba*І and *Pst*I enzymes, then the gene fragment was properly ligated into the pWB980 expression vector with the appropriate orientation under the control of P43 promoter. The ligated vector was successfully transferred into *Bacillus subtilis* WB600 using electroporation method. Several transformant clones were grown in LB agar containing 10 µg/mL kanamycin at 37 °C for 48 h, then verified by PCR, enzymatic digestion, and sequencing. Our results showed that 7 out of the 9 transformants contained plasmids with correct orientation of the gene fragment which were candidated for gene expression. Double digestion of recombinant plasmids by *Eco*RІ and *Xba*І into 0.5 kb and 4 kb fragments confirmed the correct orientation exactly (Figure 3c).

### 3.3. Expression of lysostaphin gene

**Figure 4.**
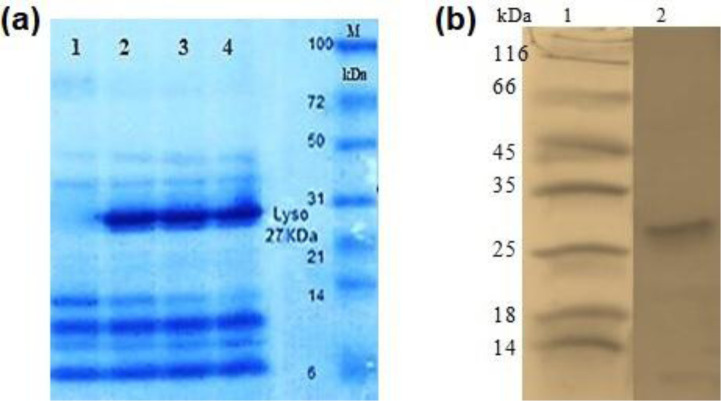
(a) Protein profile of the *B. subtilis* extracellular enzymes. Lane 1: Control proteins from *B. subtilis* cells; Lanes 2, 3, and 4: Lysostaphin protein expression in three different times (6, 8, and 12 h, respectively); Lane M: Molecular weight protein marker-Fermentas. (b) Western blotting analysis of the lysostaphin. Lane 1: Molecular weight protein marker (kDa); Lane 2: The lysostaphin protein blot

SDS-PAGE analysis of target protein showed a band corresponding to approximately 27-kDa r-lysostaphin (Figure 4a). This protein was confirmed on Western immunoblot by using HRP-conjugated Goat Anti-Rabbit IgG shown in Figure 4b. The recombinant protein concentration was estimated approximately 91 mg/L by the Bradford protein assay.

### 3.4. Enzymatic activity of r-lysostaphin

**Figure 5.**
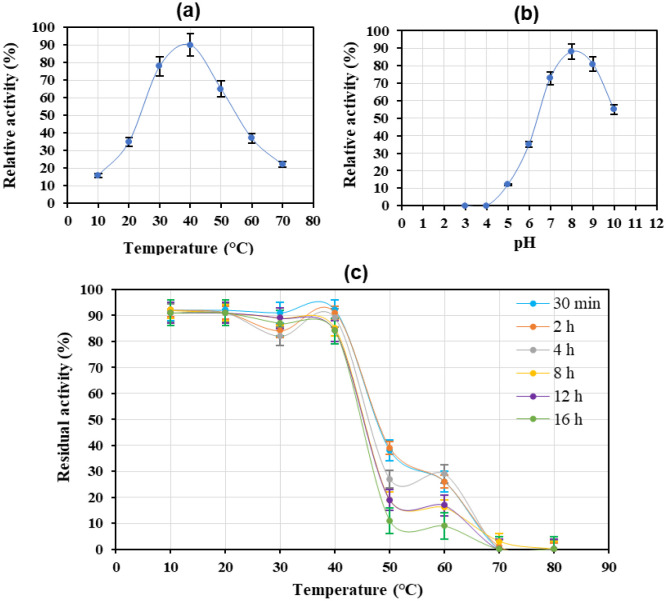
(a) Effect of temperature on r-lysostaphin activity. One U of enzyme was used for reaction in the activity test. The results have been represented as the average values of three replicates. (b): Effect of pH on r-lysostaphin activity. One U of enzyme was used for reaction in the activity test. The results have been represented as the average values of three replicates. (c): Heat residual activity (HRA) of r-lysostaphin at pH 8.0 and optimum concentration of substrate in OD_620 nm_ = 0.25

The temperature dependence of enzyme activity was assayed in the range of 20–70 °C. The optimum temperature activity of r-lysostaphin was reported 37–40 °C; however, the enzyme represented 90% of its maximum activity at 40 °C. Significantly, the r-lysostaphin displayed good thermostability by keeping about 80% of its maximum activity at 45 °C as a well-known high temperature for the denaturation of most enzymes (Figure 5a). The pH profile studies on the staphylolytic activity of r-lysostaphin in the range of 5.0–10.0 showed maximum activity at pH 8 (Figure 5b). Heat residual activity assay of r-lysostaphin ranging from 10 to 80 °C for the different time intervals of 30 min, 2, 4, 8, 12, and 16 h demonstrated that the enzyme stability was up to 40 °C. Notably, the recombinant enzyme showed good stability at 40 °C for 16 h incubation (Figure 5c).

## 4. Discussion and conclusion

In the present study, the mature lysostaphin from *S. simulans* was successfully cloned and expressed in the *B. subtilis* WB600 under the transcriptional control of the strong and regulated P43 promoter of pWB980 expression system. The pWB980 vector contains an auto-inducible P43 promoter, sacB signal sequence, multiple cloning site (MCS), and kanamycin-resistance marker derived from *B. subtilis* [19]. The P43 promoter has been previously characterized, validated, and applied in the constitutive overexpression of exogenous genes within *B. subtilis* vectors [20]. It has been shown that the P43 promoter to be recognized and active throughout the exponential and lag phases of growth due to the probable recognition of the promoter by both sigma factor 55, the major sigma factor, and sigma factor 37, the lag phase sigma factor. The signal sequence sacB allows the heterologous proteins to be secreted into the culture medium [19]. We did not add any inducers during the fermentation process of *B. subtilis* expressing r-lysostaphin due to the constitutive auto-inducible P43 promoter of pWB980 vector. We obtained extracellular secreted r-lysostaphin in the expected molecular weight of mature lysostaphin with 27 kDa. We were able to produce 91 mg/L r-lysostaphin which was an impressive concentration of recombinant protein in comparison with the previous studies [21],[22]. A recent study reported the yield of about 30 mg of r-lysostaphin per liter of the growth medium of *E. coli* BL21(DE3) in the pET-32a(+) system [23]. Previously, Recsei, PA produced approximately 150 mg mature active lysostaphin per liter of culture medium using pJP1 expression system in *Bacillus sphaericus* strain 00 [24]. In an another study, a three-fold increase in lysostaphin yield was achieved from 100 mg/L to 300 mg/L using the nisin-controlled gene expression system NICE of *Lactococcus lactis* [25]. Former approaches for lysostaphin production purified the enzyme from the crude extract of *S. simulans* [4]. The products might be contaminated by pyrogens and/or allergens. Also, the mature lysostaphin is cleaved off during the extract method process. Therefore, purity and yield of wild-type lysostaphin were very limited [21],[23]. The other methods, including ion-exchange chromatography, isoelectric focusing [5], Sephadex G-50/100 gel filtration [26], Sephacryl S-200 gel filtration chromatography [21], and intein-chitin-binding domain (CBD) system [13] have been reported for the purification of lysostaphin from the culture filtrate of *S. simulans*. Cloning and expression of the lysostaphin gene have been reported in various strains such as *E. coli* BL21(DE3) as a common host for the production of heterologous proteins [23]. However, there are some occasions where *E. coli* is not host strain of choice and alternative hosts like *B. subtilis* may become attractive [27]. Obviously, a large amount of the target protein is synthesized as insoluble form and deposited in the inclusion bodies of *E. coli* expression systems. Solubilization and refolding of the target proteins from their insoluble form lead to a major loss in specific protein activities [22]. *B. subtilis* has emerged as a highly popular expression host possessing advantages (i.e., non-pathogenicity, absence of significant codon bias, presence of secretory mechanisms, and a well-defined sporulation and germination system), and it is considered as a safe expression host, as far as pharmaceutics/therapeutics is concerned and numerous proteins have been expressed in it [28]. Interestingly, *B. subtilis* produces high levels of extracellular proteins and express them directly into the culture medium. Foreign-secreted proteins typically remain well-folded and in their biologically active form facilitating downstream processing and purifications [29]. *B. subtilis* WB600 host strain has deficient in six extracellular proteases, including nprE, aprA, epr, bpf, nprB, and mpr, which completely overcome the degradation of *B. subtilis* proteases problem [30].

This is the first report on cloning and expression of the lysostaphin enzyme gene in *B. subtilis* WB600 using pWB980 expression system, which secretes a high level of r-lysostaphin enzyme several folds rather than the production of r-lysostaphin by pET and some other T7-based expression hosts *E. coli* BL21(DE3), BL21 star(DE3), BL21-A1, T7 Express, RV308(DE3), and HMS174(DE3). The optimum temperature and pH activity of r-lysostaphin were found at 37–40 °C and 8, respectively which is in concordance to the previous reports [31],[32].

The recombinant approach from a non-pathogen, safe, and high potent organism and source of lysostaphin as well as its easy downstream processing and purification can be considered intrinsic for industrial-scale production of the valuable therapeutic staphylococcal agent. Other approaches, such as codon optimization and/or site-directed mutagenesis in the target gene sequence may be effective to overproduce the lysostaphin with a high specific activity.
